# Minutes of the Third Annual Meeting of the Alumni Association of the Chicago Medical College

**Published:** 1870-04

**Authors:** 


### MINUTES OF THE THIRD ANNUAL MEETING OF THE ALUMNI ASSOCIATION OF THE CHICAGO MEDICAL COLLEGE.

The members of this association met in the lecture room of the College, at 4½ P.M., March 22d, 1870. Dr. Julien S. Sherman, President, occupied the chair.

Minutes of previous meeting read and approved.

Interesting papers were read, from Drs. G. Wheeler Jones, Stacy Hemenway, George H. Fuller, T. G. Williams, J. L. Kitchen, and others.

The report of the committee on prize essays was then read.

*Report of Committee on Prize Essays.*

The undersigned committee, appointed to examine such essays as might be presented for the prize of $100, offered by the Alumni Association of the Chicago Medical College, respectfully report: That but two essays were placed in the hands of the committee, within the time specified by the association. One of these, bearing the motto, "Amicus Humani Generis," presents a very creditable resume of the history, varieties, diagnosis, pathology, and treatment of typhoid fever. The other, bearing the motto, "Cereless A. B.," presents a good summary of our present knowledge concerning trichinæ and trichiniasis. Both essays would do credit to their authors, as contributions to some medical periodical; but neither embodies the results of such original investigations and research as would justify your committee in recommending the award of an important prize. We, therefore, recommend that the offer of the same prize be continued another year, hoping that a more extended competition will be elicited thereby. Respectfully submitted,

N. S. DAVIS,

E. ANDREWS,

H. A. JOHNSON,

WALTER HAY.

*Chicago, March 22, 1870.*

The essays are in the hands of the Secretary, awaiting the direction of their authors.

It was moved and carried that the report be accepted, the recommendation adopted, and the committee requested to serve another year.

The annual address, by the retiring President, Dr. Sherman, was listened to with marked attention, and elicited hearty applause.

On motion of Dr. D. A. Sheffield, of Apple River, Ill., a Necrologist was elected, whose duty is to collect all the history possible, of each deceased member of the association, and shall from such data write a biographical sketch of the same, which shall be read at the first subsequent annual meeting of the association, and be published in its reports. On motion of the same, the Secretary and Necrologist were elected to hold their office during their membership in the association.

The Secretary's report showed that 37 graduates of the College were practicing their profession in Chicago.

Elections resulted in choosing the following officers:

President, John M. Woodworth, M.D., Chicago, class '62.

1st Vice-President, D. A. Sheffield, M.D., Apple River, Ill., class '67.

2d Vice-President, C. C. Reichard, M.D., Monmouth, Ill., class '70.

Secretary and Treasurer, S. A. McWilliams, M.D., Chicago, permanent.

Necrologist, Norman Bridge, M.D., Chicago, permanent.

No further business appearing, the association adjourned.

I take great pleasure in informing the Alumni that $100 is offered for a prize to that member of the Association who shall present the best essay on some medical or surgical topic. The subject of the essay to be left to the option of the competitor. All are members who have paid their yearly dues of one dollar. Any Alumnus not now a member can become one by sending his name and one dollar to the Secretary, which it is hoped every one will do. The committee on competing essays consists of N. S. Davis, M.D.; E. Andrews, M.D.; H. A. Johnson, M.

D.; and W. Hay, M.D. Each essay must be designated by a device or motto, and must be accompanied by a sealed envelope bearing the same device or motto, and containing the name and address of the author. The envelope belonging to the successful essay will be opened, and the name of the author announced, at the next annual meeting of the Association, also, at the next annual commencement of the College, in 1871. The competing essays must be sent in to the Secretary of the Association on or before the 15th February, 1871. It is expected that the prize essay, and others of marked value, will be published in pamphlet form, and furnished to members of the Association. You are earnestly urged to become a member of the Association, in full standing, by sending one dollar to the Secretary and Treasurer. S. A. McWILLIAMS,

*Permanent Secretary and Treasurer,*

166 State Street.

